# Broadband localization of light at the termination of a topological photonic waveguide

**DOI:** 10.1126/sciadv.adr9569

**Published:** 2025-04-18

**Authors:** Daniel Muis, Yandong Li, René Barczyk, Sonakshi Arora, L. Kuipers, Gennady Shvets, Ewold Verhagen

**Affiliations:** ^1^ Kavli Institute of Nanoscience, Department of Quantum Nanoscience, Delft University of Technology, 2628 CJ Delft, Netherlands.; ^2^ School of Applied and Engineering Physics, Cornell University, Ithaca, NY 14853, USA.; ^3^ Center for Nanophotonics, AMOLF, Science Park 104, 1098 XG Amsterdam, Netherlands.

## Abstract

Localized optical field enhancement enables strong light-matter interactions necessary for efficient manipulation and sensing of light. Specifically, tunable broadband energy localization in nanoscale hotspots offers many applications in nanophotonics and quantum optics. We experimentally demonstrate a mechanism for the local enhancement of electromagnetic fields based on strong suppression of backscattering. This is achieved at a designed termination of a topologically nontrivial waveguide that nearly preserves the valley degree of freedom. The symmetry origin of the valley degree of freedom prevents edge states to undergo intervalley scattering at waveguide discontinuities that obey the symmetry of the crystal. Using near-field microscopy, we reveal that this leads to strong confinement of light at the termination of a topological photonic waveguide, even without breaking reciprocity. We emphasize the importance of symmetry conservation by comparing different waveguide termination geometries, confirming that the origin of suppressed backscattering lies with the near conservation of the valley degree of freedom, and show the broad bandwidth of the effect.

## Teaser

Light is observed to localize and enhance at the termination of a topological photonic waveguide due to suppressed backscattering.

## INTRODUCTION

Manipulation of light in topological photonic platforms offers an exciting paradigm for exploring fundamental principles of light-matter interactions and developing communication technology components with new functions or improved performance (*1*, *2*). Specifically, photonic systems with nontrivial topology of their bulk eigenstates feature useful phenomena such as helical edge states (*3*–*10*). Careful tailoring of these photonic systems offers opportunities for efficient and robust molding of light on a nanophotonic chip, important for, e.g., coupling of quantum emitters (*11*), integrated photonic circuits (*12*, *13*), and microcavity lasing (*14*–*16*).

Topological photonic edge states can emerge at an interface of dielectric photonic crystals (PhCs) with broken spatial symmetry (*4*, *5*). Such broken symmetry can open a broad bandgap in which bidirectional spin- or valley-locked chiral edge states exist. These edge states are known to exhibit suppressed backscattering by spin- or valley-flipping processes at corners that obey the protecting symmetry of the crystal (*9*, *17*). This notion of reduced backscattering and robust transmission raises the intriguing question of what happens to optical energy in a system if a topological waveguide is abruptly terminated. Related to this question, in nonreciprocal waveguides, it was proposed that optical intensity could be enhanced at a waveguide termination, specifically in waveguides based on magneto-optic surface plasmon-polaritons (*18*–*24*) and on bianisotropic metamaterials (*22*). These envisioned effects have been theorized to have a topological origin (*22*), and it has been argued that their large and broadband field enhancements can benefit light-matter interactions and nonlinear optics (*25*, *26*). This suggests that reciprocal topological waveguides could also facilitate such enhancement, potentially broadening the scope of applications. It was predicted that an abruptly terminated photonic valley Hall channel can localize light in a hotspot at the termination (*27*). This topology-enabled buildup of optical energy is distinct from Anderson localization in disordered waveguides and from other conventional mechanisms for local field concentration, such as at PhC defects or other resonant nanocavities. Instead, it is related to the near conservation of the valley degree of freedom (DOF) of waves reflecting from the termination, as the minimization of intervalley overlap is expected to delay backreflection.

In this work, we experimentally demonstrate localization and enhancement of optical energy at a termination of a topological waveguide at telecom frequencies using near-field microscopy (*28*). We observe that light intensity is enhanced by an order of magnitude for a broad range of frequencies where there exist no other surface modes that can carry energy away from the termination along the interface terminating the topological waveguide. We reveal that the symmetry of the termination plays an important role in the observed effect, in line with the predicted mechanism of backscattering reduction. This mechanism, and the fact that it is not associated with a specific localized cavity mode at the termination, is elucidated by full-field and tight-binding calculations. These results thus demonstrate a new mechanism for electromagnetic field enhancement at the nanoscale, with consequently broad application potential in the control of light-matter interactions and improved manipulation and sensing of light in photonic technology.

The topological waveguide is designed by connecting two mirror-inverted valley PhCs (VPCs) (*5*). The VPCs have a rhombic unit cell that is composed of two opposite-facing triangular air holes with an inequivalence in size, which breaks inversion symmetry and therefore opens a two-dimensional photonic bandgap (*5*, *7*, *29*). Inversion symmetry breaking results in an opposite-signed Berry curvature locally concentrated at the K/K′ valleys in the Brillouin zone. Connecting two VPCs that have distinct topologies, a valley-dependent edge state emerges, which emulates the quantum valley Hall effect and is protected against intervalley scattering at defects that conserve the *C*_3_ crystalline symmetry (*8*, *17*). Reciprocity guarantees that two edge states associated with the opposite valley DOF propagate in opposite directions. As long as the edge state dispersion lies below the light line, light is confined to the dielectric PhC slab.

**Fig. 1.**
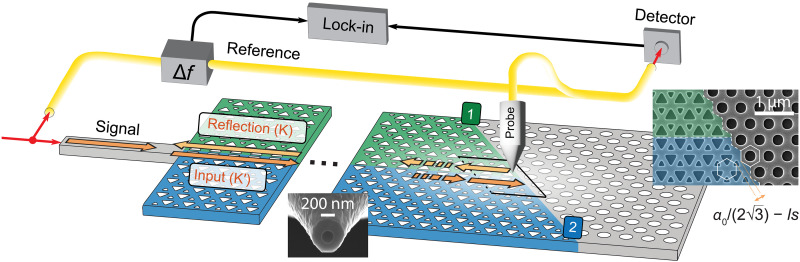
Schematic of the experiment. High-level diagram of the near-field scanning optical microscope setup and the topological waveguide terminated by a trivial PhC. The colors (green and blue) depict two mirrored nontrivial PhCs and the trivial PhC (gray) at the termination. Orange arrows with different shades depict forward- and backward-propagating edge states in K′ and K valleys. Infrared light enters via a silicon ridge waveguide from the side of the sample and propagates robustly in the topological waveguide until it scatters at the termination. Light may couple into the output ports (1) and (2). A near-field probe raster-scans above the surface and detects a fraction of the components of the in-plane electric field. The detected signal is recombined with the reference branch, which is given a 60-kHz offset in frequency, resulting in a beating signal that is read out by lock-in amplifiers. Insets show scanning electron microscopy (SEM) images of a near-field probe and a symmetry-protected zigzag termination. The distance between the VPCs and the trivial PhC depends on the lattice shift (*ls*) parameter and affects the frequency range at which surface modes appear in the photonic dispersion diagram.

The topological waveguide is abruptly terminated by an insulating trivial PhC with a two-dimensional photonic bandgap that prohibits further propagation of the edge state. This termination results in a triple junction of three inequivalent interfaces as shown in Fig. 1. The two interfaces between the VPCs and the PhC can in principle support trivial surface states. These states are not expected to span the entire VPC gap, as the difference in the valley Chern numbers of the VPC and the PhC is 1/2. Instead, each interface exhibits a frequency gap where no propagating states exist. We consider of particular interest the regime where the frequency gaps of both interfaces overlap. This frequency regime is bound by surface modes of both interfaces, and we hence refer to it as the surface mode gap. In the surface mode gap, the edge state in this specific VPC-VPC interface design has a relatively high group velocity. Therefore, scattering because of intervalley conversion at random fabrication disorder, which can be substantial in slow-light waveguides (*30*), is negligible. Operating at a frequency in this gap, light that is incident from the valley Hall waveguide toward the termination can only scatter out of plane (to free space radiation) or back into the topological waveguide at the termination. However, backscattering requires a flip of the valley index, which is suppressed at a suitably designed termination (*27*). We emphasize that the suppression mechanism does not mean that light is not reflected: The reciprocal nature of the system and the spatially localized perturbation formed by the termination guarantee that it does. At suitable termination, the suppression however means that light is reflected with a significant delay. We investigate the resulting signature of suppressed backscattering on broadband optical enhancement at the termination.

## RESULTS

### Experimental observation of optical energy enhancement

**Fig. 2.**
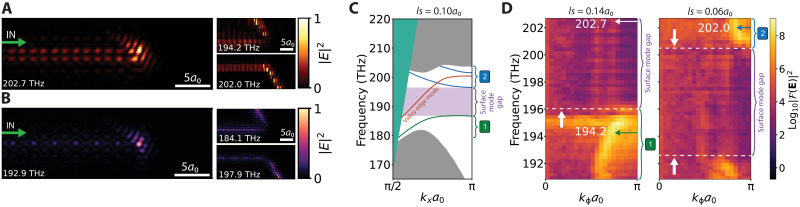
Optical energy enhancement at the zigzag termination. (**A**) Experimental measurement of the near-field in-plane electric field at a lattice shift of 0.14*a*_0_, normalized to the maximum of the scan at corresponding frequency (bottom left corner). Optical energy fed by the valley topological waveguide localizes at the termination for frequencies in the surface mode gap. Panels on the right show that for frequencies outside the surface mode gap, the valley edge mode couples to the trivial surface modes, resulting in propagation along the terminating interface. The upper (lower) panel depicts this propagation at a lattice shift of 0.14*a*_0_ (0.06*a*_0_). (**B**) Near-field in-plane electric field at a lattice shift of 0.10*a*_0_ in simulation. (**C**) Simulation of the photonic band diagram for a lattice shift of 0.10*a*_0_ showing the surface mode gap in between the surface modes (green and blue lines). The valley edge state (orange line) spans the entire surface mode gap, shown here in the vicinity of valley K′ ≡ 2π/3. Bulk modes are located in the solid gray areas. Indicated by the solid green area is the region above the light line, where radiative losses occur. The offset with experiment is ~7 THz. (**D**) Photonic band diagram in experiment for lattice shifts of 0.14*a*_0_ and 0.06*a*_0_ at the VPC-PhC interface. For a larger lattice shift, the surface mode bands shift up in frequency because of a stronger spatial confinement at the termination. Dashed lines indicate the limits of the surface mode gap, and arrows depict the frequencies of the experimental near-field scans in (A). Bulk modes from the VPCs appear below 192.5 THz.

To observe the spatial distribution of light near the termination of a topological waveguide, we use scanning-probe near-field microscopy, as shown schematically in Fig. 1. A heterodyne detection technique allows for phase-resolved detection with high signal-to-noise ratio (see Materials and Methods). Infrared light enters the VPC-VPC interface via a silicon ridge waveguide from the side of a silicon-on-insulator (SOI) sample. As the forward-propagating edge state (at the K′ valley in the *k*-space; dark orange arrow in Fig. 1) encounters the termination, it can couple to one of the trivial surface modes that propagates along the VPC-PhC interface (output ports labeled 1 or 2). A near-field probe that is raster scanned above the surface picks up a fraction of the evanescent in-plane electric field components with subwavelength resolution, limited by the 194-nm aperture of the aluminum-coated near-field probe. The raster scan allows imaging the intensity of light in the PhC as a function of position in the two-dimensional space at a height of ~20 nm above the surface. The intensity is derived directly from the electric field components and is shown in the real space for three different laser frequencies in Fig. 2A. At a laser frequency of 202.7 THz, the edge state is seen to propagate through the topological waveguide until it terminates upon encountering the trivial PhC. At the termination, the edge state strongly localizes with the enhancement of the local field energy in a spot with a spatial extent of ~520 nm.

The two smaller panels on the right side of Fig. 2A depict scans from the same area but at different laser frequencies. Moreover, for a reason that is explained below, the image labeled “202.0 THz” was obtained on a sample with a slightly different design, distinguished by a small shift of the trivial PhC lattice with respect to the VPC crystals. The right inset of Fig. 1 illustrates the terminating interface and depicts the shift between the two distinct lattices, quantified by a parameter called “lattice shift” (*ls*) (see the Supplementary Materials for the detailed definition). In the inset of Fig. 1, the lattice shift is 0.00*a*_0_. The image labeled “202.0 THz” in Fig. 2A was taken for a lattice shift of 0.06*a*_0_ and the other two for a lattice shift of 0.14*a*_0_. As we see below, a smaller lattice shift lowers the frequency at which specific behavior is observed. In the images on the right side, we observe that an edge state propagates along the VPC-PhC interface away from the termination. The light propagates into either port (1) for the lowest of the two frequencies (194.2 THz, lattice shift of 0.14*a*_0_) or port (2) for the higher frequency (202.0 THz, lattice shift of 0.06*a*_0_). The outgoing ports correspond to an acute or obtuse angle, respectively, with respect to the direction of the incident VPC-VPC edge state (see the Supplementary Materials for extended plots over the entire sample area). The two characteristic phenomena, namely optical energy localization and the out-coupling to one of the trivial surface modes, agree with simulation results (Fig. 2B).

Figure 2 (A and B) shows that the behavior of scattering at the termination, as well as the observed localization, depends pronouncedly on the optical frequency. The frequency regimes associated with the different phenomena can be understood from the COMSOL-simulated dispersion diagram shown in Fig. 2C. The diagram depicts the frequency dependence of the guided mode wave vectors along the direction of propagation in the incident VPC-VPC edge (orange line) and in both output ports (1) and (2) (green and blue lines, respectively). Wave vectors are shown in the range [π/(2*a*_0_), π/*a*_0_], which is equivalent to the positive part of the first Brillouin zone that lies below the light line. We note that the waveguide modes and bandgaps are consistently found at lower frequencies in simulations than in the experiments, likely due to slight geometrical deviations of the fabricated sample. The absolute frequencies in the simulated panel C should therefore be compared to the simulated images in panel B but not to the experimental images in panel A. Within the topological bandgap but out of the surface mode gap, incoming light from the valley edge mode can be carried away from the termination in port (1) at low frequencies and in port (2) at high frequencies. When operating at frequencies in the surface mode gap, no propagating surface states are supported by output ports (1) and (2), while the VPC-VPC interface does support the topological edge state.

Experimentally, we probe the frequency dependence by executing raster scans of the near-field probe for a range of laser frequencies. By using a two-dimensional Fourier transform of a region containing the VPC-PhC interface and projecting the intensity normal to the direction of the interface, we retrieve the wave vectors of light propagating along the VPC-PhC interface, i.e., along output ports (1) and (2). The results are experimental dispersion diagrams (Fig. 2D), showing a ~12-THz window that is accessible with our tunable laser. The two panels are taken on structures with different lattice shift values of 0.14*a*_0_ and 0.06*a*_0_. At frequencies below the surface mode gap, a surface mode propagating along port (1) exists at the terminating interface with *ls* = 0.14*a*_0_ (Fig. 2D, left panel). For a smaller lattice shift, *ls* = 0.06*a*_0_, all surface mode dispersion curves shift to lower frequencies, and a surface mode propagating along port (2) emerges within our observation window (Fig. 2D, right panel). This overall downshift of all surface mode frequencies is because a slight lattice shift, which corresponds to a broader spatial gap between the VPC and the PhC lattices, increases the effective index of guided waves along the terminating interface. We note that for *ls* = 0.06*a*_0_, the surface mode along port (1) is shifted to such low frequencies that it overlaps with the bulk modes of the VPC. Thus, both surface modes along the output ports labeled in Figs. 1 and 2C can be observed, with their precise frequency tunable through the lattice shift parameter. Besides the aforementioned offset in a frequency of ~7 THz of experiment to simulation, the results are consistent. From the two dispersion diagrams we retrieve for lattice shifts of 0.14*a*_0_ and 0.06*a*_0_, it becomes clear that a broadband surface mode gap exists where the incident VPC-VPC edge state cannot couple to either upper or lower propagating surface states. Instead, the edge state may either scatter back or scatter in the out-of-plane direction to free-space radiation, resulting in a loss of the on-chip optical energy. Backscattering is, however, symmetry dependent and can be suppressed by a termination with a particular orientation. This suppression results in an accumulation of optical field energy that we observe in Fig. 2A. We again clarify that even if a significant fraction of the incident light is eventually reflected into the backward direction, the suppression leads to a delay and the associated optical hotspot.

### Role of symmetry in protection and field enhancement

When a valley edge mode encounters the termination, its evanescent tails penetrate into the two VPC bulks and are affected by the symmetry of the termination. As this can affect the rate of backscattering, we investigate the correlation between the observed localization and the symmetry of the termination. Along terminations with different geometries, the overlap between the forward-propagating (K′ valley) and backscattered (K valley) modes is substantially different. Some unique choices of the termination can greatly suppress this overlap and hence reduce the rate of backscattering (see the Supplementary Materials for detailed theoretical derivation). We distinguish between zigzag terminations, corresponding to π/3 or 2π/3 angles between the VPC-PhC and VPC-VPC interfaces, and armchair terminations, corresponding to the π/2 angle. We reveal that zigzag terminations largely suppress the overlap, while armchair terminations feature a considerable overlap. This mathematical conclusion can be intuitively understood as follows: A zigzag termination maximally follows the symmetry of the VPC lattice and thus conserves the valley DOF to a great degree, exploiting the topologically nontrivial nature of the VPC crystals. In contrast, an armchair termination strongly breaks the crystal symmetry that protects the VPC-VPC states from scattering efficiently. While a certain degree of symmetry breaking is unavoidable for any shape or termination, and light can thus eventually scatter back, the rate at which this happens thus depends strongly on the symmetry of the termination in the topological crystal (*27*). Last, we note that the finite size of the termination in the three-dimensional slab setting means that some scattering to free space is possible. There is in principle no reason to assume that the amount of free-space scattering as a result of this defect is notably different for zigzag and armchair terminations.

**Fig. 3.**
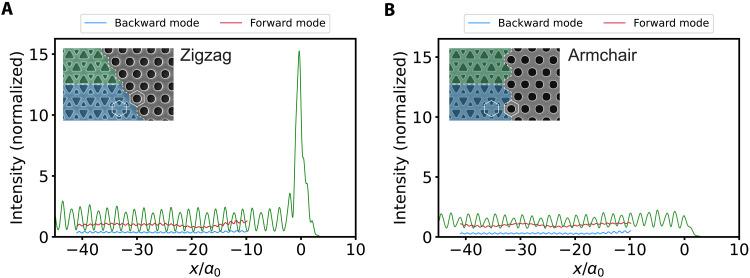
Optical energy enhancement for the two terminations. Experimental measurement of the intensity along the length of the topological waveguide, normalized to the intensity of the forward-propagating mode. Light from the laser is coupled in at a frequency of 200.0 THz. (**A**) The topological waveguide is terminated at position *x*/*a*_0_ = 0. Energy localization only occurs at the zigzag termination, shown for a lattice shift of 0.06*a*_0_, which nearly conserves the valley DOF and suppresses backscattering. The spatial confinement of the enhancement peak is ~3*a*_0_. (**B**) The armchair termination, shown for a lattice shift of 0.10*a*_0_, shows a uniform energy distribution in the waveguide and no enhancement. Segments of the forward and backward components of the electromagnetic wave retrieved using filters in the *k*-space are shown in the middle of the topological waveguide. Insets: SEM images of zigzag and armchair VPC-PhC interfaces. Note that the zigzag SEM image is actually for *ls* = 0.00*a*_0_.

To study the effect of termination geometry experimentally, we probe the electromagnetic near-field intensity near the end of the waveguide for samples with either zigzag or armchair terminations. First, we experimentally confirm the optical energy localization at a zigzag termination. The green curve in Fig. 3A shows the intensity as a function of position along the VPC-VPC waveguide, which we obtain by integrating the intensity in a two-dimensional scan over a transversal width of two lattice constants across the center of the waveguide. We observe for both the zigzag termination in Fig. 3A and the armchair termination in Fig. 3B an oscillatory behavior in intensity along the majority of the VPC-VPC waveguide. This is the result of interference between forward- and backward-propagating modes for all Bloch harmonics. Our phase-sensitive heterodyne detection scheme allows separating the intensities of forward and backward waves by applying a wave vector filter on the Fourier-transformed fields around the respective Bloch components (*17*). The inverse Fourier transform using only the *k_x_* components that correspond to the forward (backward) propagating mode then returns the individual contribution to the intensity by that mode, shown by the red (blue) curve in a translationally invariant part of the topological waveguide. We refer to section S4 for a detailed explanation of the formalism behind the forward/backward separation. We observe a substantial intensity of backward propagation for both the zigzag and armchair terminations, for any frequency within the surface mode gap, and for different values of lattice shift, as shown in fig. S3. As the observation frequency resides in the surface mode gap (see Fig. 2D and fig. S4D), there can be no energy propagation along the VPC-PhC interfaces. Light does, however, extensively scatter out of plane as a consequence of symmetry breaking at the termination, explaining a reduced intensity of the backward-propagating mode. Furthermore, the intensity after being reflected from a zigzag or armchair termination is approximately the same (see fig. S5A), indicating that the out-coupled energy to the free space does not depend strongly on the type of termination.

The local intensity at the zigzag termination is observed to be 15 times larger than the average intensity of the forward-propagating mode in the waveguide in a sharply localized hotspot. In stark contrast, the armchair termination shows no enhancement, meaning that all energy is immediately reflected back. Simulations confirm that optical energy is strongly enhanced only at the zigzag termination (see fig. S6). These observations can be theoretically understood by considering a cavity that is formed by a finite length of valley-Hall waveguide between two terminations. In figs. S7 and S8, we demonstrate that all eigenmodes of this closed system can be understood as Fabry-Pérot modes, which are caused by reflections of guided waves at the ends of the cavity. All mode frequencies depend on the length of the valley-Hall channel, i.e., the topological waveguide, consistent with their identification as Fabry-Pérot modes. This observation concludes that there is no excitation of a local resonant cavity mode at the termination, which would appear with a frequency independent of length. The observed localization is instead a broadband effect on the guided waves impinging on the termination. By studying the field distributions of the Fabry-Pérot modes in the doubly terminated waveguides, we recognize that light intensity is enhanced in all modes near the zigzag termination. In stark contrast, no such localization is observed in the eigenmode profiles for armchair terminations, which show trivial Fabry-Pérot standing waves with homogeneous energy distribution. We show that this strong symmetry dependence, which we already recognized in the experiments in Fig. 3, is seen in both full-field simulations (fig. S7) and tight-binding calculations (fig. S8). The latter shows that this phenomenon is not specific to electromagnetic boundary effects at a termination but instead stems purely from lattice symmetry. Last, we also note that spectral mode density for short waveguide lengths is enhanced for zigzag terminations, in line with the notion that light is expected to be stored for considerable time at the terminations. We thus confirm theoretically, and demonstrate in experiment, that light localizes at a termination because of suppressed backscattering, which is strongly dependent on its symmetry.

### Broadband frequency dependence of enhancement

**Fig. 4.**
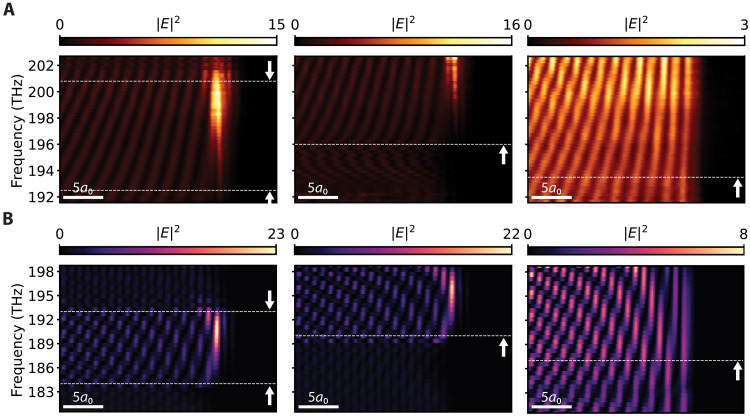
Broadband field enhancement. Experimental measurement (**A**) and simulation (**B**) of the field intensity inside the valley topological waveguide as functions of frequency. Plots are normalized by the average intensity of the forward-propagating mode in the center of the waveguide. From left to right: zigzag termination with lattice shifts of 0.06*a*_0_ and 0.14*a*_0_ and armchair termination with a lattice shift of 0.10*a*_0_. At zigzag terminations, the optical energy is up to 15 times larger than in the middle of the waveguide, whereas at the armchair termination, the optical energy is uniform along the entire waveguide. Horizontal dashed lines show the limits of the surface mode gap. The upper limit may appear out of the frequency range provided by the laser.

The experiments in the previous section studied the localized field enhancement for a particular laser frequency of 200.0 THz within the surface mode gap. We next investigate the enhancement in a broad frequency range. Figure 4 shows a map of intensity as a function of laser frequency and position along the waveguide. Each horizontal line is obtained in a similar way as the green integrated intensity line in Fig. 3. Three different terminations with varying lattice shift are compared: two zigzag types and one armchair type. This comparison confirms that the enhancement of field intensity only occurs at a zigzag termination and not at an armchair termination. Figure S9 shows three more armchair terminations with distinct lattice shifts, revealing that enhancement is always absent for any armchair termination and thus is independent of the local effective refractive index at the termination. We observe that for zigzag terminations, the enhancement is present over ~6 THz and is the largest for frequencies within the surface mode gap. Its magnitude, which is the ratio between the intensity of light at the termination and the average intensity of forward-propagating light in the waveguide, varies with frequency and reaches a maximum just below the upper limit of the gap (white dashed line in Fig. 4A). Figure S5 (B and C) depicts the spectral shape of this broadband peak. Here, the role of the lattice shift as a tuning parameter once again becomes clear: An increase in the lattice shift narrows down the silicon gap between the VPCs and the trivial PhC, decreasing the local effective refractive index and increasing the frequencies of the surface mode bands. As a result, the enhancement peak being enclosed by the surface modes also shifts to higher frequencies. However, the maximum magnitude of enhancement is independent of the lattice shift and thus is independent of the local amount of dielectric material near the termination.

A possible interpretation of both the frequency dependence and the sharply defined spatial extent of the localized enhancement could be sought in the involvement of evanescent states of the VPC-PhC interface at the termination. Within the surface mode gap, the incident edge state cannot excite propagating states with a real wave vector along the VPC-PhC interfaces. However, a continuum of evanescent states with an imaginary wave vector along the interfaces can be expected to exist in the gap (*31*), which could be excited given appropriate wave function overlap with the incident waveguide field. These states cannot propagate but could radiate into either the free space or backward-propagating mode. In this interpretation, the suppressed scattering could be thought of as being assisted by compatible evanescent states at the interfaces, confined to the termination, that contribute to the buildup of local optical intensity there. As the overlap of incident and evanescent states would depend on the intricate details of their local fields, which are generally frequency dependent, their excitation amplitude can therefore vary slowly with frequency. In principle, evanescent modes could also exist at the armchair-terminated waveguide but with an overlap that may be substantially different from a zigzag-terminated waveguide. The absence of any localization suggests that the intervalley scattering is much stronger and the edge states instead couple directly to the backward-propagating mode. The substantial difference in localization at this different symmetry hints that the coupling to evanescent states is negligible. This is a remarkable distinction in scattering behavior between the zigzag- and armchair-terminated waveguides. We note that this possible viewpoint of storage in local evanescent states does not provide an alternative explanation to the general effect of localization at a topological waveguide termination that we report, but it could provide a route to understanding the details of spatial and spectral extent of the intensity enhancement in future work.

We note that localization of optical energy is also observed experimentally at the zigzag termination for frequencies slightly above the upper gap edge. Within the same viewpoint, we note that for those frequencies, upper surface modes could be excited as long as there is a finite overlap with the wave function of the edge state. As such states will have low group velocity near the band edge, they are expected to contribute to local field enhancement. For the lower surface modes, the overlap may be insufficient, as no enhancement is observed here. Only in the case of sufficient wave function overlap and for the modes with a group velocity substantially lower than the group velocity of the edge state, modes can become confined at the termination and in the output ports. In some cases, this results in an intensity in the output port that is higher than inside the topological waveguide, as seen in Fig. 2 (A and B). The magnitude of intensity in the output port however depends on how much light scatters out of plane or reflects back to the topological waveguide. Figure S4 shows the optical energy at an armchair termination and shows no high levels of intensity in the output ports. Light is simply allowed to backscatter, resulting in a reduced amount of energy flowing to the output ports. The heterogeneous distribution of energy at distinct terminations further underscores the pivotal role of the near conservation of the valley DOF.

## DISCUSSION

Using VPCs as a topological photonic platform, we demonstrate that light can be substantially localized at a termination of a reciprocal topological waveguide. Only for terminations that approximately conserve the valley DOF, reflection is suppressed strongly enough to result in localization. We observe a broadband peak of the optical intensity in the frequency range of the surface mode gap at a zigzag termination of the waveguide. For frequencies above the surface mode bandgap, less prominent localization is attributed to the wave function overlap between the edge state and the upper surface modes with a low group velocity. Measurements of the local intensity at an armchair termination show no enhancement at all, confirming the role of the symmetry-protected topology in the enhancement mechanism. We provide a theoretical description explaining the origin of the suppressed backscattering and also confirm the experimentally observed light enhancement using simulations.

This experimental observation of broadband light enhancement at a termination of a topological photonic waveguide is an example of a broader class of systems that could facilitate field enhancement because of suppressed backscattering (*26*). Topologically nontrivial waveguides with a termination protected by lattice symmetry pave the way for the development of novel devices exploiting the potential of enhanced light-matter interactions. Further research should explore the possible magnitude of broadband enhancement and the role of local design features, such as the lattice shift and the choice of the terminating crystal. It would be interesting to study how the localization effect could be maximized and to correlate the local field enhancement with measurements of the delay time of backscattering, for example, using pulsed optical measurements or other techniques that exploit the large bandwidth of the effect. More broadly, the demonstrated paradigm of optical energy storage and localization, in combination with robust guiding and manipulation of light, presents opportunities to on-chip photonic technology.

## MATERIALS AND METHODS

### Device fabrication

We fabricate a PhC slab on a SOI platform with a 220-nm–thick silicon layer on a 3-μm buried oxide layer. First, a positive electron-beam resist with a thickness of 240 nm (AR-P 6200.09) is spin coated. Then, the PhC design is patterned into the resist using electron-beam lithography on a Raith Voyager with 50-kV beam exposure. The electron-beam resist is developed in pentyl acetate/*O*-xylene/methyl isobutyl ketone:isopropyl alcohol (9:1)/isopropanol, and the SOI chip subsequently undergoes reactive-ion etching in HBr and O_2_.

Next, a Suss MABA6 Mask Aligner patterns the photosensitive resist AZ1518 to define a selective wet-etching window on the PhC. After development with AZ400K:H_2_O, the buried-oxide layer is partially removed by wet etching in a 1:2 solution of 40% hydrofluoric acid and deionized water for 20 min. The sample is then subjected to critical point drying to obtain free-standing PhC membranes (*32*). Last, to enable incoupling from the side, the SOI chip is cleaved on the end facets of the Si-ridge waveguides that extend to the PhCs. The Si-ridge waveguides are tapered down to support only the fundamental transverse electric (TE) mode at the VPC end facet. The ridge waveguides adiabatically transition to the VPC waveguide inside the lattice, enabling better index matching for efficient incoupling (*33*).

To account for fabrication imperfections that shift the operating frequencies of the devices, we fabricate triangular PhC lattices with three different lattice constants *a*_0_ = [449 nm, 494 nm, 539 nm]. In the VPCs, a rhombic unit cell contains two triangular holes of side lengths *s*_1_ = 0.65*a*_0_ and *s*_2_ = 0.38*a*_0_. A domain wall is created along VPC1 and VPC2 by applying a parity operation along the spatial *y*-coordinate. In the photonic bandgap material, a rhombic unit cell contains a single circular hole with a radius *r* = 0.27*a*_0_.

### Near-field microscopy setup

The near-field probe is fabricated by heated pulling of a single mode optical silica fiber using a P-2000 micropipette puller. Subsequently, the tips are etched in buffered oxide 7:1 for 20 min to ensure a wide opening angle of the aperture. The tips are coated homogeneously using a Temescal FC-20349 evaporator with first ~1.5-nm chromium for improved adhesion and then ~150-nm aluminum. The aluminum coating protects against radiative light loss at considerable distances from the tip. The aluminum at the tip is then partly milled away by focused ion beam etching (Helios G4Cx) to create an aperture of 194 nm.

Probes are attached to one prong of a Farnell AB38T quartz tuning fork with a resonance of 32.768 kHz. The tuning fork has a *Q*-factor of ~700 and is soldered to a custom-made chip that drives the tuning fork and reads out its oscillating frequency. The chip is then connected to a piezo dither block, which raster scans the probe above the PhC while being controlled by a shear force feedback loop via a digital lock-in amplifier (Zurich Instruments HF2LI), keeping the tip at an expected height of ~20 nm above the PhC surface.

### Laser system

The light source deployed in the experiment is a continuous wave-tunable laser (Santec TSL-710), which is operable in the infrared frequency range (1480 to 1640 nm). In a heterodyne detection scheme, the beam is split into a signal and a reference path by a polarizing beam splitter and the frequency of the light in the reference path is shifted by 60 kHz using two acousto-optic modulators. The signal path is then coupled into the PhC ridge waveguide using a high–numerical aperture (0.8) microscope objective. A near-field probe picks up a fraction of the evanescent fields above the sample. Subsequently, signal and reference paths are recombined, which form a beating signal at 60 kHz, facilitating the extraction of the electric field components including phase resolution with a high signal-to-noise ratio via a pair of lock-in amplifiers (Stanford Research Systems SR830).

### Finite element frequency-domain simulation

All numerical simulations of the photonic structure are performed using the finite element frequency-domain software COMSOL Multiphysics. Because of the high cost of three-dimensional simulations on the computation resource, we simplify the simulation by leveraging the symmetry property of the structure. The suspended Si slab is mirror symmetric about the midheight plane (z=t/2, where *t* is the slab thickness). Therefore, all electromagnetic modes can be classified as transverse electric (TE) polarized [$E\left(z=t/2\right)⊥\hat{z}$] and transverse magnetic (TM) polarized [$E\left(z=t/2\right)∥\hat{z}$] according to their field distributions at that plane (*31*). We focus on TE-polarized modes. By setting the midheight plane of the slab as a perfect magnetic conductor, we simulate only half of the entire geometry, and only TE-polarized solutions are allowed. Above the slab (ε_slab_ = 12.11), an air domain (ε_air_ = 1) with a thickness of 3*a*_0_ is included in the simulation domain. Except for the midheight plane of the slab, all other boundaries are set using the scattering boundary condition.

## Supplementary Materials

### This PDF file includes:

Sections S1 to S5 Figs. S1 to S9 Table S1 References
