# Supplementary material for: Broadband localization of light at the termination of a topological photonic waveguide
Source: Sci Adv. 2025 Apr 18;11(16):eadr9569. doi: 10.1126/sciadv.adr9569 (PMC12007573; doi:10.1126/sciadv.adr9569)
Supplement: Supplementary file 1 — Sections S1 to S5 Figs. S1 to S9 Table S1 References [file sciadv.adr9569_sm.pdf]

Science Advances
AAAS

Supplementary Materials for

Broadband localization of light at the termination of a topological photonic waveguide

Daniel Muis *et al.*

Corresponding author: L. Kuipers, l.kuipers@tudelft.nl; Gennady Shvets, gs656@cornell.edu; Ewold Verhagen, e.verhagen@amolf.nl

Sci. Adv. **11**, eadr9569 (2025)
DOI: 10.1126/sciadv.adr9569

This PDF file includes:

Sections S1 to S5
Figs. S1 to S9
Table S1
References

Section S1. Design of the termination

This section describes the design of the terminating interface and explains how a lattice shift (ls) value quantifies the distance between the VPCs and the trivial PhC, as a specific design parameter. The termination of the topological waveguide described in this paper follow two different arrangements, namely zigzag and armchair. The zigzag termination is oriented in the direction of the rhombic primitive cells of the VPC, at an angle of $\pi/3, 2\pi/3$. Fig. S1A depicts areas of an SEM scan, orthogonal to the terminating interface and with a width of a_0. The area with the green shade and labelled (1) is the VPC bulk above the interface, where the larger triangular hole of the VPC primitive cell lies closest to a circular hole of the trivial PhC. The area with the blue shade and labelled (2) is the VPC bulk below the interface, where the smaller triangular hole of the VPC primitive cell lies closest to a circular hole. The VPCs and the trivial PhC have the same honeycomb lattice structure. The distance between the VPC lattice and the trivial PhC lattice, specifically between the corners of the hexagonal cells, is quantified as $a_0/\left(2\sqrt{3}\right) - \text{ls}$, which is equivalent above and below the VPC-VPC interface. However, because the larger triangle takes up slightly more space than the smaller triangle, the effective refractive index is different at the interfaces. This results in different frequency ranges of the surface modes as described in the main text and shown in Fig. 2. The lattice shift parameter slightly shifts the position of the hexagonal cell of the circular hole so that the VPC-PhC interface gap becomes narrower or broader. Effectively, the ls parameter describes a shift of the entire lattice of holes in the PhC region. This has no effect on light dispersion in the crystal, but slightly modifies the termination. Adjusting ls affects the effective refractive index at the terminating interface and consequently the frequency at which a surface mode exists. We note that any perturbation of the termination geometry could affect the dispersion of the surface modes, and changing the position of the holes is just a straightforward way to achieve this. Fig. S1B shows an armchair terminating interface which has an equal distance between the VPC and trivial

PhC holes everywhere along the interface. This distance is quantified as $0.5a_0 - ls$, and is also adjustable by the same lattice shift value (ls). The effective refractive index is equivalent everywhere along the terminating interface, resulting in the existence of only one surface mode, as shown in Fig. S4.

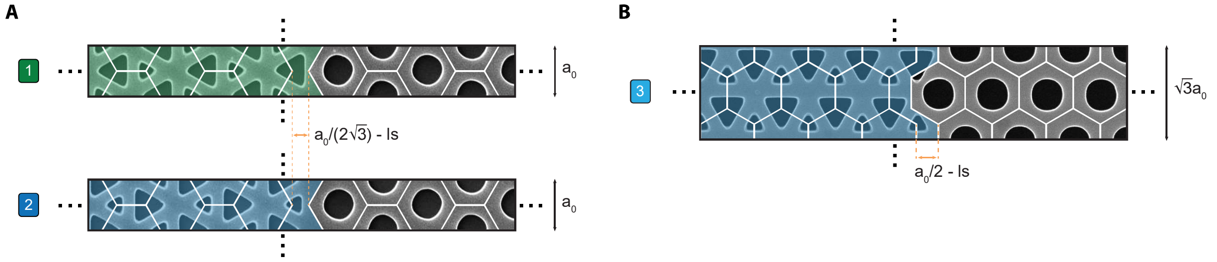

Fig. S1. Lattice shift at the terminating interface. **(A)** SEM image of a zigzag terminating interface above (green) and below (blue) the VPC-VPC interface. The distance between the corners of the hexagonal cells of the VPC lattice and the trivial PhC lattice is quantified by an assigned lattice shift value (ls). A larger lattice shift corresponds to less space between the lattices. Here ls = $0.00a_0$. **(B)** The armchair terminating interface is equivalent everywhere along the terminating interface. In the example shown, ls = $0.10a_0$, shifting the trivial PhC lattice slightly to the left.

Section S2. Extended near-field real space scans

The main text shows zoomed-in regions ($27a_0$ by $8a_0$) of the near-field intensity close to the termination. Fig. S2 shows the original scans which are much larger ($89a_0$ by $67a_0$) and almost capture the entire photonic crystal. The larger raster scans do not capture the position where light enters the photonic crystal because scattering is usually very high here. The field distribution of light in the topological waveguide and in the output ports is clearly visible.

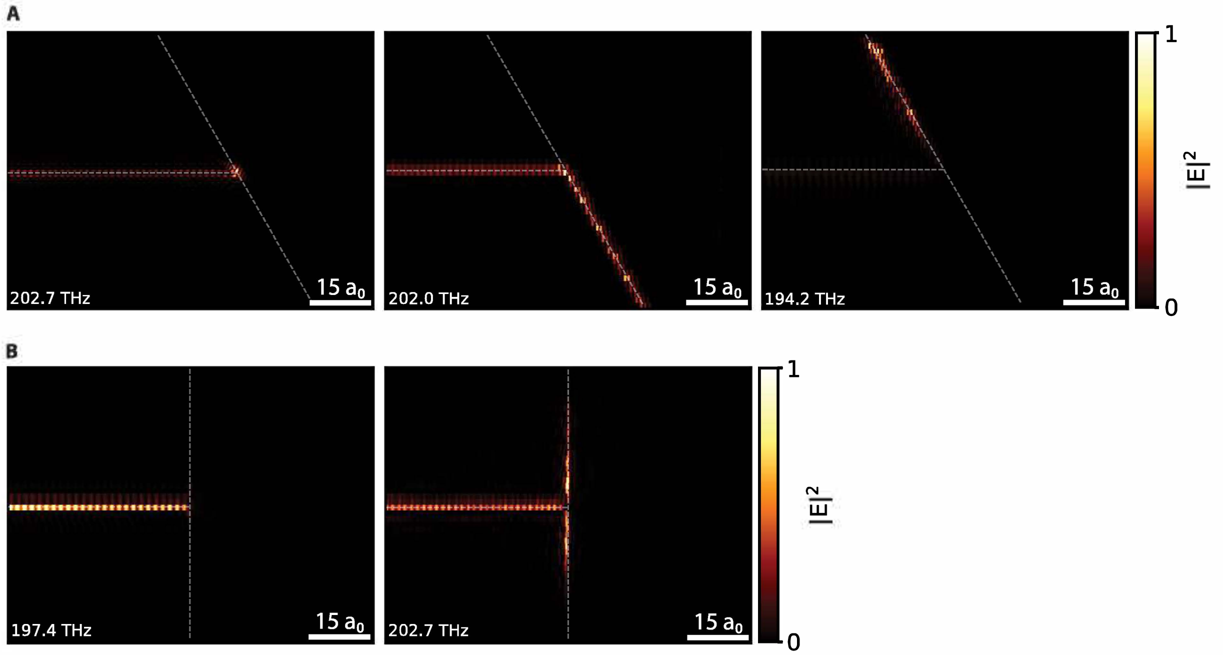

Fig. S2. Extended real space scans. Experimental measurements of the near-field in-plane electric field intensity, normalized to the maximum of the scan at corresponding frequency. **(A)** Optical energy fed by the valley topological waveguide localizes at the zigzag termination for a frequency in the surface mode gap, shown for ls $0.14a_0$ in the left panel. For a particular range of frequencies outside the surface mode gap the edge state couples to a surface mode and light propagates along the VPC-PhC interface to the edge of the PhC, shown for ls $0.06a_0$ and ls $0.14a_0$ in the center and right panel, respectively. **(B)** Optical energy fed by the valley topological waveguide is uniform and does not localize at the armchair termination for a frequency in the surface mode gap, shown for ls $0.10a_0$ in the left panel. For particular frequencies, surface modes exist to which the edge state can couple, resulting in light propagating in both ways along the VPC-PhC interface, shown for ls $0.20a_0$ in the right panel. Dashed grey lines illustrate interfaces of zigzag and armchair terminations.

Section S3. Evaluation of the wave function overlap along zigzag and armchair terminations

(The following evaluation is rewritten based on the Supplementary Materials of Ref. (*27*), which extends the derivation in the Supplementary Materials of Ref. (*5*))

In this section, we analyze how the quantum valley Hall (QVH) edge modes scatter when encountering zigzag and armchair terminations. We demonstrate that the QVH edge mode weakly backscatters on a zigzag termination and strongly backscatters on an armchair one. The backscattering coefficient, which is quantified as the overlap between the two time-reversal conjugated modes (i.e., with opposite valley indices) $\Psi^*_{\mathbf{K}'}\Psi_{\mathbf{K}}$, shows a unique dependence on κ, the inverse of the edge mode's transverse decay length.

The QVH edge mode can be written as $\Psi_{\mathbf{K}} = e^{i\mathbf{K}\cdot\mathbf{r}}u_{\mathbf{K}}(\mathbf{r})e^{-\kappa|y|}$. The overlap between the counterpropagating edge modes is $\Psi^*_{\mathbf{K}'}\Psi_{\mathbf{K}} = e^{i(\mathbf{K}-\mathbf{K}')\cdot\mathbf{r}}u^*_{\mathbf{K}'}(\mathbf{r})u_{\mathbf{K}}(\mathbf{r})e^{-2\kappa|y|}$, where $\mathbf{K} = (4\pi/(3\mathrm{a}_0), 0)$ and $\mathbf{K}' = (-4\pi/(3\mathrm{a}_0), 0)$. $u_{K'(K)}$ are periodic functions with the same periodicity of the lattice. Therefore, $u_{\mathbf{K}'}u_{\mathbf{K}}$ also shares the same periodicity as the lattice, and the overlap can be written as,

$$\Psi^*_{\mathbf{K}'}\Psi_{\mathbf{K}} = e^{i\frac{8\pi}{3\mathrm{a}_0}x}e^{-2\kappa|y|}\sum_{m,n} a_{m,n}e^{i(m\mathbf{b}_1+n\mathbf{b}_2)\cdot\mathbf{r}} \tag{S1}$$

where $\mathbf{b}_{1,2}$ are the reciprocal lattice vectors, $\mathbf{b}_{1,2} = \frac{2\pi}{\mathrm{a}_0}\left(1, \pm 1/\sqrt{3}\right)$.

Then, we consider a termination with the same periodicity as the lattice. We can write the spatial distribution of the termination as a product of the components perpendicular and parallel to the termination direction, $A(\mathbf{r}_\perp)\left(\sum_l b_l e^{il\frac{2\pi}{\mathrm{a}_0}\mathbf{r}_\parallel}\right)$. The overlap of $\Psi^*_{\mathbf{K}'}\Psi_{\mathbf{K}}$ integrated along the

terminating interface direction ($\mathbf{r}_\parallel$, also represented as the θ-direction), is

$$
\begin{aligned}
&A(\mathbf{r}_\perp)\int_{-\infty}^{\infty} dr_\parallel \sum_l b_l e^{il\frac{2\pi}{a_0}\mathbf{r}_\parallel}\psi^*_{\mathbf{K}'}\psi_{\mathbf{K}} \\
=&A(\mathbf{r}_\perp)\sum_{l,m,n} a_{m,n}b_l \int_{-\infty}^{\infty} dr_\parallel e^{il\frac{2\pi}{a_0}r_\parallel + i\frac{8\pi}{3a_0}x + i(m\mathbf{b}_1+n\mathbf{b}_2)\cdot\mathbf{r}_\parallel - 2\kappa|y|} \\
=&A(\mathbf{r}_\perp)\sum_{l,m,n} a_{m,n}b_l \int_{-\infty}^{\infty} dr_\parallel e^{i\frac{2\pi}{a_0}[l+\left(\frac{4}{3}+m+n\right)\cos\theta + \sqrt{1/3}(m-n)\sin\theta]r_\parallel - 2\kappa\sin\theta|r_\parallel|} \\
=&A(\mathbf{r}_\perp)\sum_{l,m,n} a_{m,n}b_l \int_{-\infty}^{0} dr_\parallel \left(e^{i\frac{2\pi}{a_0}[l+\left(\frac{4}{3}+m+n\right)\cos\theta + \sqrt{1/3}(m-n)\sin\theta]r_\parallel - 2\kappa\sin\theta|r_\parallel|}\right. \\
&\qquad\qquad\qquad\qquad\left. + e^{-i\frac{2\pi}{a_0}[l+\left(\frac{4}{3}+m+n\right)\cos\theta + \sqrt{1/3}(m-n)\sin\theta]r_\parallel - 2\kappa\sin\theta|r_\parallel|)}\right) \\
=&A(\mathbf{r}_\perp)\sum_{l,m,n} a_{m,n}b_l \frac{4\kappa\sin\theta}{4\kappa^2(\sin\theta)^2 + \frac{4\pi^2}{a_0^2}\left[l+\left(\frac{4}{3}+m+n\right)\cos\theta + \sqrt{1/3}(m-n)\sin\theta\right]^2},
\end{aligned}
\tag{S2}
$$

where θ represents the direction of the termination: $\theta = 0, \pi/3, 2\pi/3$ correspond to the zigzag termination; $\theta = \pi/6, \pi/2, 5\pi/6$ correspond to the armchair termination. We shall see that it is the term $[l + \left(\frac{4}{3} + m + n\right)\cos\theta + \frac{1}{\sqrt{3}}(m - n)\sin\theta]$ in the denominator that determines the behavior of the overlap integral. For convenience, we represent this term with $c_{l,m,n}(\theta)$. Possible values of $c_{l,m,n}(\theta)$ for different terminations along the six different directions are listed in Table S1.

We notice that, with an armchair termination, $c_{l,m,n}(\theta)$ is allowed to attain a zero value while, with a zigzag termination, it is not. In the small κ limit ($2\kappa \ll 2\pi/a_0$), the quantity $4\kappa\sin\theta/\left[4\kappa^2(\sin\theta)^2 + (4\pi^2/a_0^2)\left(c_{l,m,n}(\theta)\right)^2\right]$ is negligible as long as $c_{l,m,n}(\theta) \neq 0$.

According to Table S1, for zigzag terminations, the coefficient $c_{l,m,n}$ cannot be zero because a fractional number always remains. However, for armchair terminations, the coefficient $c_{l,m,n}$ can attain zero. With the specific combination of $\{l, m, n\}$ that makes $c_{l,m,n} = 0$, the quantity $4\kappa\sin\theta/\left[4\kappa^2(\sin\theta)^2 + (4\pi^2/a_0^2)\left(c_{l,m,n}(\theta)\right)^2\right]$ diverges and makes the $\Psi^*_{\mathbf{K}'}\Psi_{\mathbf{K}}$ overlap diverge.

At last, we discuss the validity of the small κ limit. $\kappa = \Delta/(2v)$, where Δ is the topological band gap width and v is the slope of the Dirac cone before opening the band gap. For the

microwave QVH photonic crystal in Refs. (*27, 34*), $\Delta \approx 0.0398(2\pi c/\mathrm{a}_0)$ and $v \approx 0.42c$, so $1/\kappa \approx 3.36\mathrm{a}_0$, and the small κ condition is satisfied.

θ	termination shape	$c_{l,m,n}(\theta) \equiv l + \left(\frac{4}{3} + m + n\right)\cos\theta + \frac{1}{\sqrt{3}}(m-n)\sin\theta$
0	zigzag	$l + \frac{4}{3} + m + n$
$\frac{\pi}{6}$	armchair	$\frac{\sqrt{3}}{6}(4m + 2n + 4) + l$
$\frac{\pi}{3}$	zigzag	$m + l + \frac{2}{3}$
$\frac{\pi}{2}$	armchair	$l + \frac{1}{\sqrt{3}}(m-n)$
$\frac{2\pi}{3}$	zigzag	$-n + l - \frac{2}{3}$
$\frac{5\pi}{6}$	armchair	$\frac{\sqrt{3}}{6}(-2m - 4n - 4) + l$

Table S1. Values of $\mathrm{c}_{\mathrm{l,m,n}}(\theta)$ for different terminations.

Section S4. Numerical recipe for separating the forward propagating and backward propagating components

This section describes how the forward- and backward propagating modes are separated in simulation and experiment. The intensity of the forward propagating mode is repeatedly used for normalization of the optical energy enhancement.

In simulation

For convenience, we use $\psi(\mathbf{r}, f)$ to represent the six-component electromagnetic field of the structure that is simulated using COMSOL at frequency f,

$$\psi(\mathbf{r}, f) \equiv \left[E_x(\mathbf{r}, f), E_y(\mathbf{r}, f), E_z(\mathbf{r}, f), H_x(\mathbf{r}, f), H_y(\mathbf{r}, f), H_z(\mathbf{r}, f)\right]^T. \qquad \text{(S3)}$$

In this section, we explain how to separate the forward $(+\hat{x})$-propagating and the backward $(-\hat{x})$-propagating components. First, we convert the field to the $\hat{x}$-directional momentum space (the k_x-space) using Fourier transform,

$$\psi(k_x, y, z, f) \equiv \mathcal{F}\{\psi(x, y, z, f)\} = \int_{-\infty}^{\infty} \psi(x, y, z, f) e^{-ik_x x}\, dx. \qquad \text{(S4)}$$

The integration is over the entire simulation domain.

$\psi(k_x, y, z, f)$ is symmetric about $k_x = 0$ (within the surface mode gap) because all the input energy is reflected, and the forward and backward components are identical in amplitude.

Due to the finiteness of the simulation domain, $|\psi(k_x, y, z, f)|$ approaches zero when $k_x \to \pm\infty$. We identify that the nonzero part of $\psi(k_x, y, z, f)$ dominates in the range $-2\pi/\mathrm{a}_0 < k_x < 2\pi/\mathrm{a}_0$. Therefore, the integration out of that range is discarded when calculating the inverse Fourier transform.

The $\mathbf{K}'$ valley ($k_x = (-2/3 + 2N)\pi/\mathrm{a}_0$) corresponds to forward propagation; the $\mathbf{K}$ valley ($k_x = (2/3 + 2N)\pi/\mathrm{a}_0$) corresponds to backward propagation, where $N \in \mathbb{Z}$. Therefore, we partition the range $-2\pi/\mathrm{a}_0 < k_x < 2\pi/\mathrm{a}_0$ into two parts,

$$\text{toward right (TR)}: -\pi/\mathrm{a}_0 < k_x < 0, \quad \pi/\mathrm{a}_0 < k_x < 2\pi/\mathrm{a}_0,$$
$$\text{toward left (TL)}: -2\pi/\mathrm{a}_0 < k_x < -\pi/\mathrm{a}_0, \quad 0 < k_x < \pi/\mathrm{a}_0.$$

The forward propagating component, $\psi^{\mathrm{TR}}(\mathbf{r}, f)$, and the backward propagating component, $\psi^{\mathrm{TL}}(\mathbf{r}, f)$, are calculated by inverse-Fourier-transforming $\psi(k_x, y, z, f)$ in the two k_x partitions, accordingly,

$$\begin{aligned} \psi^{\mathrm{TR}}(x,y,z,f) &= \frac{1}{2\pi}\int_{-\pi/\mathrm{a}_0}^{0} \psi(k_x,y,z,f)e^{ik_x x}\,dk_x + \frac{1}{2\pi}\int_{\pi/\mathrm{a}_0}^{2\pi/\mathrm{a}_0} \psi(k_x,y,z,f)e^{ik_x x}\,dk_x, \\ \psi^{\mathrm{TL}}(x,y,z,f) &= \frac{1}{2\pi}\int_{-2\pi/\mathrm{a}_0}^{\pi/\mathrm{a}_0} \psi(k_x,y,z,f)e^{ik_x x}\,dk_x + \frac{1}{2\pi}\int_{0}^{\pi/\mathrm{a}_0} \psi(k_x,y,z,f)e^{ik_x x}\,dk_x. \end{aligned} \tag{S5}$$

Normalization. To compare the two structures, the waveguides with zigzag and armchair terminations, we normalize the electromagnetic field such that the input electromagnetic energy in the two setups is identical,

$$avg(\mathrm{S}_x^{TR,zig}) = avg(\mathrm{S}_x^{TR,arm}), \tag{S6}$$

where $S_x = \mathrm{Re}(E_y H_z^* - E_z H_y^*)/2$ is the time-averaged $\hat{x}$-directional Poynting vector. Here the average, $avg(\cdot)$, is taken over a waveguide segment away from the source and the termination.

In experiment

A near-field probe picks up a fraction of the evanescent electric fields above the surface and retrieves all the spatial frequencies in the PhC at a sub-wavelength scale. Having access to these spatial frequencies, we can use a two-dimensional Fourier transform to obtain all wavevectors of light in the crystal for a single laser frequency. In two-dimensional reciprocal space we then observe all the (k_x, k_y) for which modes are found. An integration along k_y then retrieves a distribution of intensity for wavevectors k_x, which is in the propagation direction of the topological waveguide. By executing the near-field raster scan for all frequencies that the laser provides and for each frequency retrieving the wavevectors k_x we can reconstruct dispersion diagrams as shown in Fig. S3. These diagrams show the dispersion of modes in valleys K and K' in the first Brillouin zone as well as the dispersion of all higher order Bloch harmonics. The slope of the dispersion indicates the sign of the group velocity $v_g = d\omega/dk$. A positive group velocity is indicative of a forward propagating mode while a negative group velocity refers to a backward propagating mode. We select an individual mode by selecting a specific interval of k_x wavevectors. Subsequently we perform an inverse Fourier transform to real-space and obtain the real space electric field intensity in the topological waveguide.

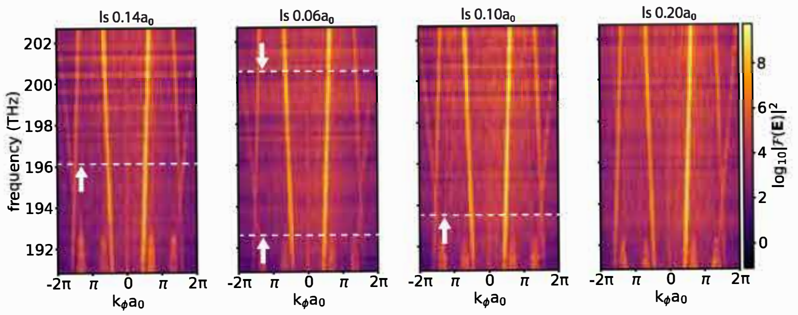

Fig. S3. Experimental dispersion diagrams of the topological waveguide. Photonic band diagrams at the VPC-VPC interface showing the first two Brillouin zones show notable amplitude of the back-reflected wave in the waveguide. An intensity offset between the forward and backward propagating wave is observed, indicating non-unity reflectance due to out-of-plane scattering in all cases. The two left diagrams have zigzag terminations and correspond to the surface dispersion diagrams of Fig. 2D. The two right diagrams have armchair terminations and correspond to the surface dispersion diagrams of Fig. S4D. For both termination types, and for different lattice shift values, a certain amount of intensity is found for the backward propagating wave, which confirms the eventual backscattering of light into the waveguide as a result from the inevitable symmetry perturbation by the trivial PhC.

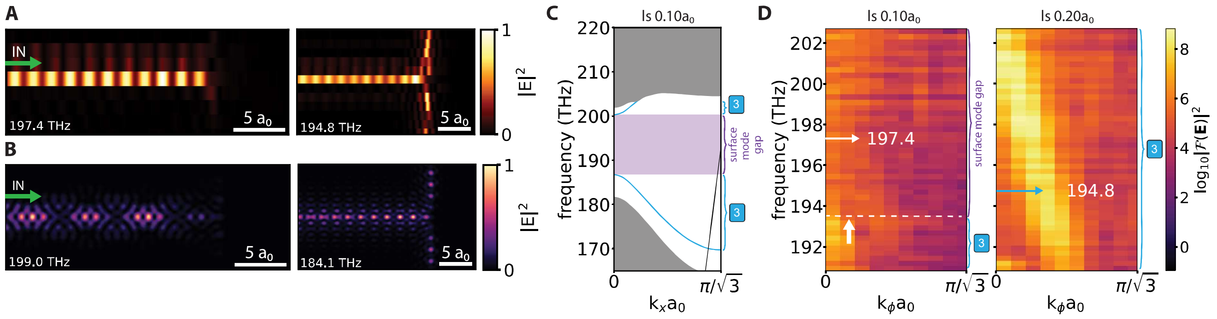

Fig. S4. Optical energy at an armchair termination. (A) Experimental measurement of the near-field in-plane electric field at an armchair termination, normalized to the maximum of the scan at corresponding frequency (bottom left corner). Optical energy fed by the valley topological waveguide does not localize at the termination. The right figure shows scattering of the valley edge mode to the trivial surface mode for frequencies outside the surface mode gap resulting in propagation along the terminating interface in both directions. The left (right) panel has a lattice shift of $0.10a_0$ ($0.20a_0$). **(B)** Near-field in-plane electric field at a lattice shift of $0.10a_0$ in simulation. **(C)** Simulation of the photonic band diagram for a lattice shift of $0.10a_0$ showing the surface mode gap and the dispersion of the surface mode (cyan line). Bulk modes are located in the solid grey area. Almost the entire diagram shows modes below the light line, meaning that radiative losses occur. The offset with experiment is $\sim$7 THz. **(D)** Experimental measurement of the photonic band diagram, for a lattice shift of $0.10a_0$ and $0.20a_0$ at the VPC-PhC interface. For a larger lattice shift the surface mode bands shift up in frequency due to a stronger spatial confinement at the termination. Arrows depict the frequencies of the scans in **(A).** Dashed lines indicate the limits of the surface mode gap. Bulk modes from the VPCs appear below 192.5 THz.

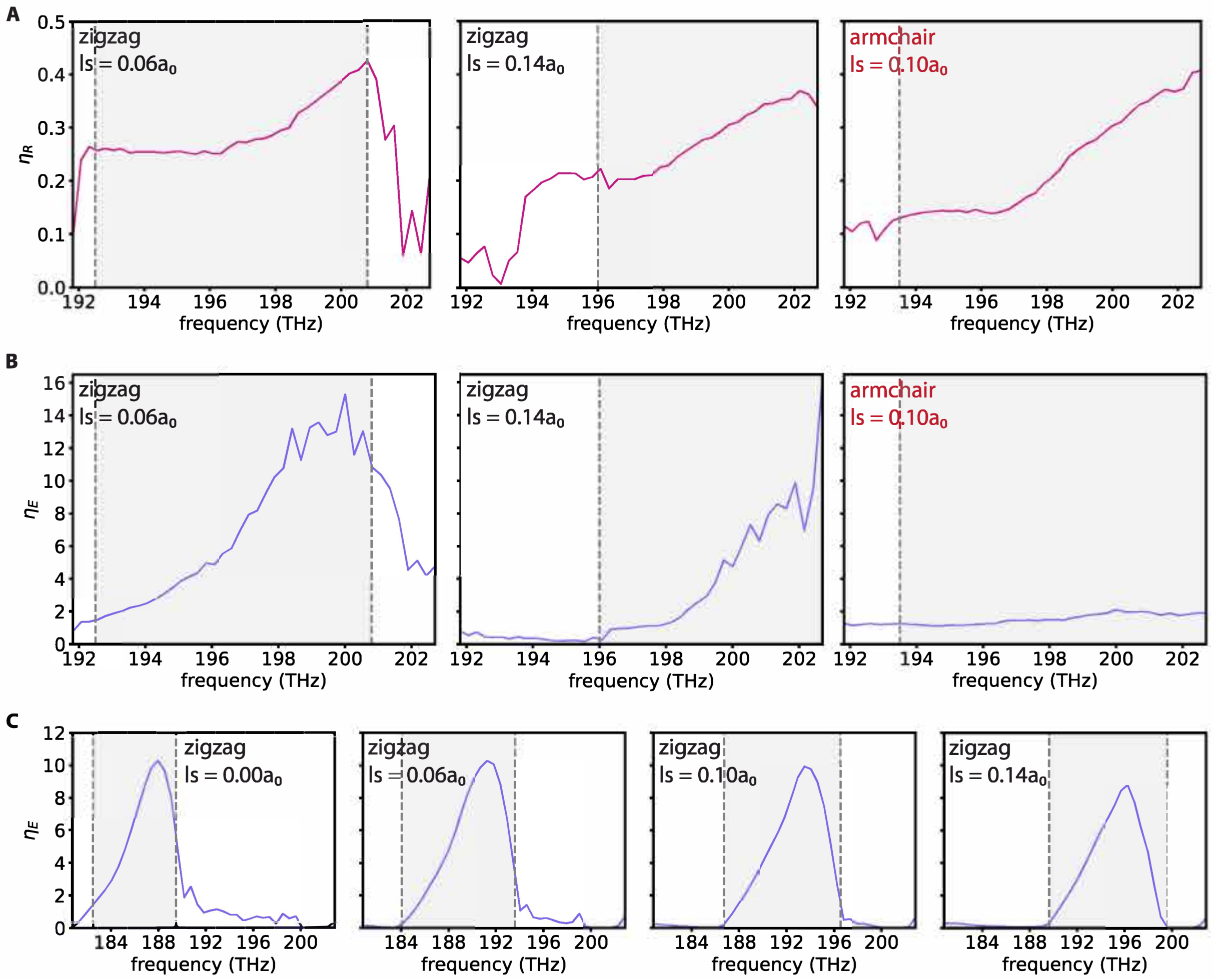

Fig. S5. Reflection and enhancement coefficients. Reflection **(A)** and enhancement **(B)** coefficients in experiment as a function of frequency for terminations with different geometries. The termination geometry and lattice shift are indicated in each plot in the upper left corner. The reflection coefficient is given as the ratio between intensity of the average backward and forward propagating mode $\eta_R = I_{s-}/I_{s+}$. We observe similar magnitudes of reflection for both armchair and zigzag terminations, with remaining differences likely attributable to the different far-field scattering properties of the respective local termination geometries. Increasing reflection coefficients above $\sim$197 THz are accounted to the transition of the edge state past the

light line. Below 197 THz, the edge state lies above the light line and can easily couple to free-space at the termination. As the group velocity of the edge state is comparable to that of the light line, the transition is broadband. The enhancement coefficient in experiment is given as the ratio between the maximum intensity at the termination and the intensity of the average forward propagating mode $\eta_E = I_{termination,\ max}/I_{s+}$. **(C)** shows the simulated enhancement coefficient for zigzag terminations, given as the ratio between the integrated intensity over a polygon at the termination and the integrated intensity of the average forward propagating mode over a polygon region around the waveguide. The broadband enhancement reaches it maximum just below the upper surface modes and shifts in frequency with changing lattice shift. The solid grey area depicts the broadband surface mode gap. The peak enhancement is seen to remain approximately equal for different lattice shifts, showing that it is not related to the local amount of dielectric material.

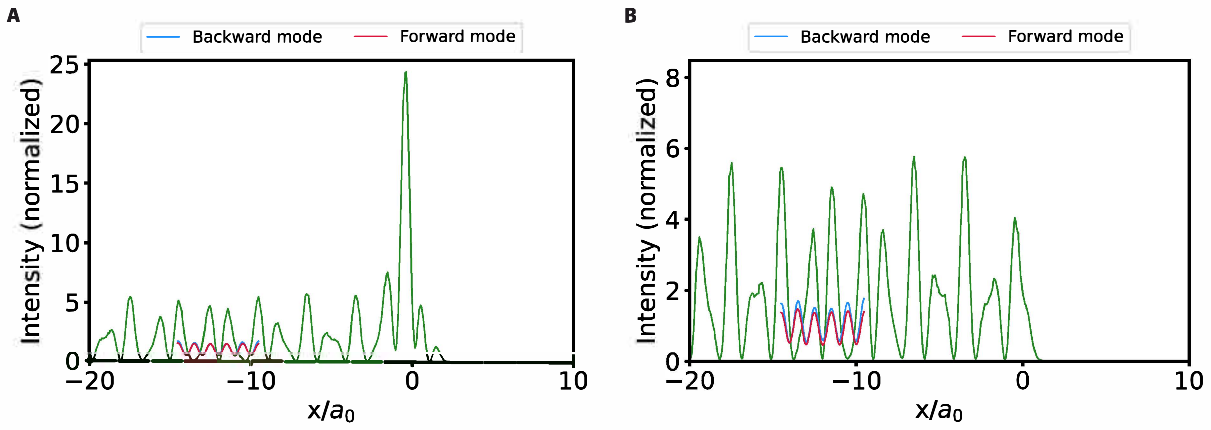

Fig. S6. Simulation of the optical energy enhancement for the two terminations. Optical energy along the length of the topological waveguide, normalized to the intensity of the forward propagating mode. Light from the source has a frequency of 191.0 THz. **(A)** The topological waveguide is terminated at position $x/a_0 = 0$. Energy localization only occurs at the zigzag termination, shown for lattice shift $0.06a_0$, which nearly conserves the valley DOF and suppresses backscattering. **(B)** The armchair termination, shown for lattice shift $0.10a_0$, shows a uniform energy distribution in the waveguide. The simulation domain is smaller than the experimental sample, due to the requirement for large computational resources for 3D simulations. Segments of the forward- and backward-component of the electromagnetic wave are shown in the middle of the topological waveguide. We remark that these simulations were performed with scattering boundary conditions. As these do not guarantee to absorb all outgoing waves, these simulations cannot be used to quantify the amount of out-of-plane scattering. Indeed, the observed amplitude of the backwards wave is larger than that in experiment, where light suffers losses due to out-of-plane scattering.

Section S5. Theoretical framework for localization in finite-length waveguides

In this section we provide additional motivation for the unique mechanism of localization that we describe in this work, and its distinction from conventional field concentration mechanisms in photonic crystal defects or other resonant nanocavities. Concentration by exciting a conventional cavity mode resonantly can in principle occur at discrete frequencies in finite systems, for example in photonic crystals. We demonstrate with two theoretical approaches that such a mode does not exist in our system.

We consider a finite length of valley-Hall waveguide which is terminated at both ends with a zigzag and armchair symmetry, respectively. We note that this is of course different from our experiments, which concern an open waveguide geometry with only one termination. We can nonetheless gain valuable insight from studying the eigenmodes of the closed system, which importantly features well-defined boundary conditions. The finite-element simulation results in Figs. S7A and S7B show that in such a finite-length waveguide, there is always a discrete set of electromagnetic modes within the band gap. It is expected that the finite-length waveguide supports a set of Fabry-Pérot modes due to the reflections of guided waves at the ends. The number of modes in the bandgap, and their discrete mode frequencies, depend on the length of the topological waveguide. If, in addition to such Fabry-Pérot waveguide modes, a localized defect mode would exist at either termination, this would occur at a frequency that is independent of the length of the waveguide. Such a mode is not observed in the spectra, neither for the zigzag-terminated waveguide (Fig. S7A), nor for armchair terminations (Fig. S7B). This confirms the absence of a localized cavity mode at the termination.

We do recognize that the zigzag-terminated system contains a higher spectral density of modes than the armchair-terminated system, in particular for short waveguide lengths. This can be understood from a non-uniform group velocity of light in the waveguide: At a zigzag

termination light is expected to undergo a time delay, as theoretically predicted in (*27*), which effectively increases the local mode density at the termination. Moreover, it is seen that at sufficiently long waveguides, the eigenmodes are distributed over the entire bandgap with equivalent separations, thus approaching the broadband spectrum of a waveguide. The difference between the zigzag and armchair termination becomes strikingly evident from the field distribution of the eigenmodes. Figs. S7C and S7D show that for the zigzag termination, the eigenmode fields are locally enhanced near the termination, for any mode in the surface mode gap, while at armchair terminations the distribution is a trivial standing wave with uniform intensity distribution. Again, this shows that the localization effect acts on the waves in the topological waveguide, and is therefore an inherently broadband effect that cannot be associated with a localized cavity mode at the termination.

We note that this unique field distribution with localization only at zigzag terminations stems from the lattice symmetry along the termination and is not an electromagnetic boundary effect specific to the photonic crystal termination. We confirm this through a tight-binding model, which does not include characteristics of light, such as polarization, and only supports a scalar wave. Figs. S8A and S8B show the eigenmode frequencies in a tight-binding model of a finite-length waveguide with two terminations. On a honeycomb lattice with nearest-neighbour coupling strength $t = 1$, staggered on-site potentials $\epsilon = 0.2$ are implemented for the two quantum valley Hall bulks (*35*). The signs of the on-site potentials of the two bulks are opposite, creating a valley Hall channel along the interface. The results are consistent with Figs. S7A and S7B. The field profiles in Figs. S8C and S8D confirm again that localization is only observed at the zigzag termination.

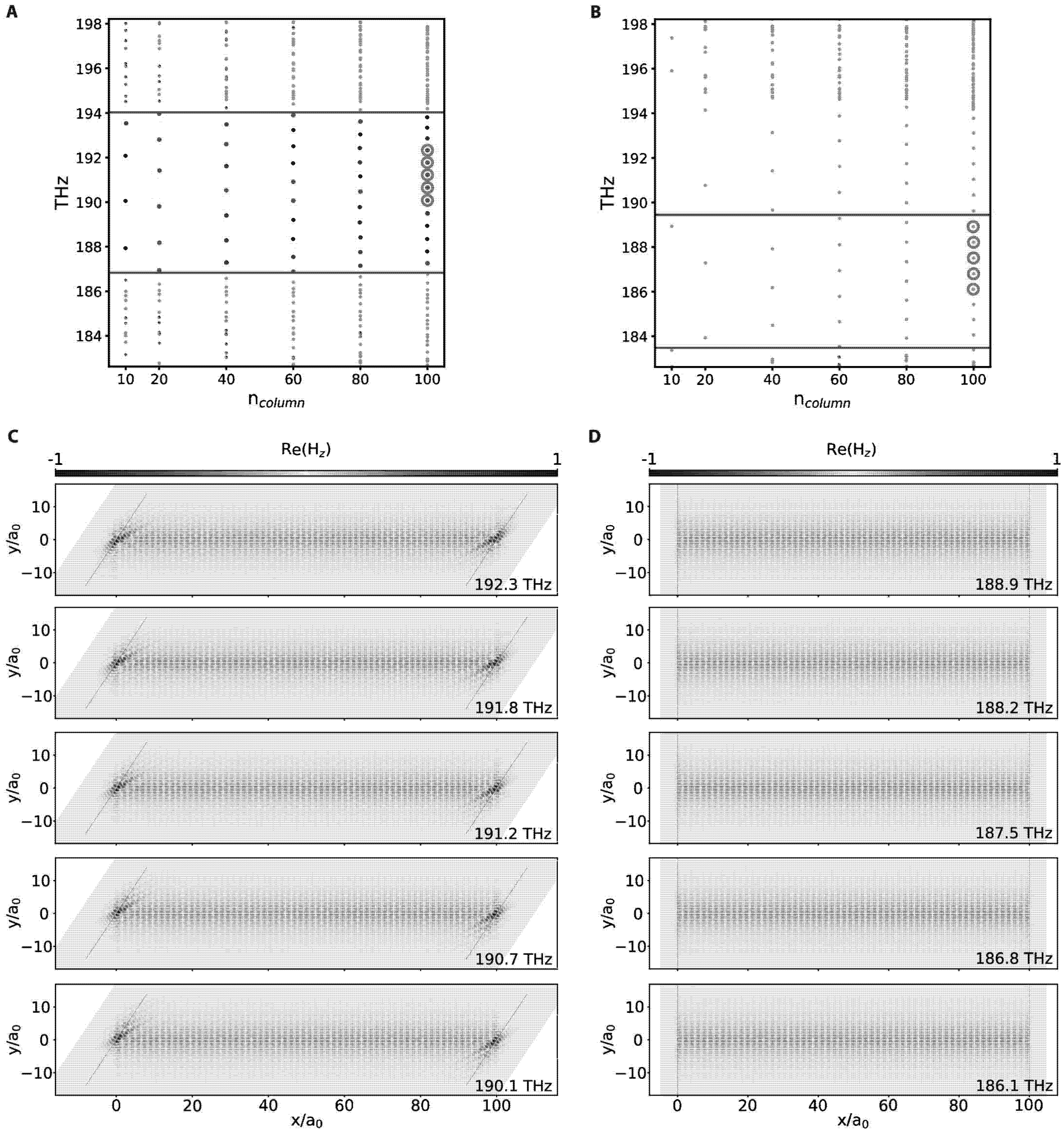

Fig. S7. Spectral dispersion and electromagnetic fields of the modes in finite-length waveguides. COMSOL-simulated eigenmode frequencies in a finite valley Hall topological waveguide between two terminations, of either zigzag type **(A)** or armchair type **(B)**, as a func-

tion of the waveguide length $a_0 n_{\mathrm{column}}$. There always exists a set of discrete Fabry-Pérot eigenmodes in the surface mode gap, whose edges are indicated by the two horizontal red lines. All mode frequencies change with the length of the waveguide, as expected for Fabry-Pérot resonances. Blue dots indicate that the field localizes at the terminations. The electromagnetic field distribution shows that the symmetry of the termination is key to localization: At zigzag terminations **(C)** all eigenmodes feature localized fields near the termination, while at armchair terminations **(D)** this effect does not show at the waveguide terminations. The example field distribution is shown in panels **(C)** and **(D)** for five frequencies at a cavity length of $x/a_0 = 100$, also indicated with green circles in plots **(A)** and **(B)**.

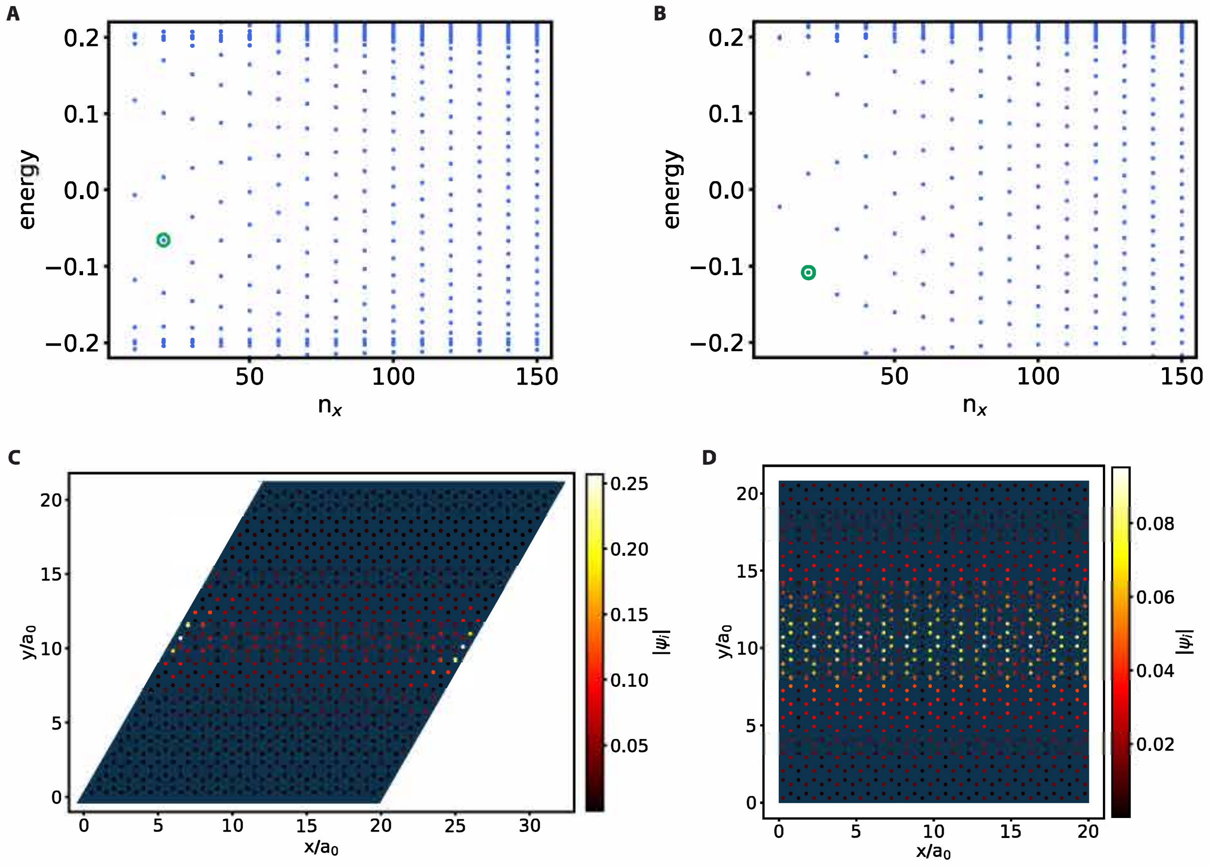

Fig. S8. Tight-binding calculation of finite waveguide spectra and mode profiles. Tight-binding model calculations showing the eigenmode energies in a waveguide of length n_x between two terminations of either zigzag type **(A)** or armchair type **(B)**. The finite waveguide always supports discrete modes, and the mode frequencies vary with the length of the topological channel, as expected for Fabry-Pérot resonances. At zigzag terminations **(C)**, the field profile always shows high amplitude at the termination, while at armchair terminations **(D)**, the field always distributes uniformly. The field distribution is shown for a solution that is indicated by a green circle in plots **(A)** and **(B)**.

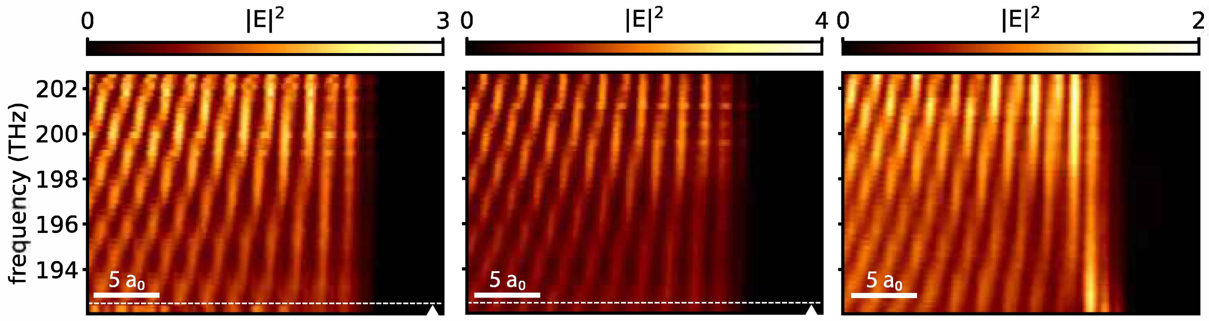

Fig. S9. Absence of enhancement at armchair terminations. Experimental measurement of the field intensity inside the valley topological waveguide as function of frequency. Plots are normalized by the average intensity of the forward propagating mode in the centre of the waveguide. At armchair terminations, the optical energy is uniform along the entire waveguide and shows no enhancement. From left to right: Armchair termination with lattice shift -0.10a$_0$, 0.00a$_0$ and 0.20a$_0$. Horizontal dashed lines show the limits of the surface mode gap. Limits may appear out of the frequency range provided by the laser. Lattice shift 0.20a$_0$ has no gap in the frequency range shown.

REFERENCES AND NOTES

1. L. Lu, J. D. Joannopoulos, M. Soljačić, Topological photonics. *Nat. Photonics* **8**, 821–829 (2014).

2. T. Ozawa, H. M. Price, A. Amo, N. Goldman, M. Hafezi, L. Lu, M. C. Rechtsman, D. Schuster, J. Simon, O. Zilberberg, I. Carusotto, Topological photonics. *Rev. Mod. Phys.* **91**, 015006 (2019).

3. A. B. Khanikaev, S. Hossein Mousavi, W.-K. Tse, M. Kargarian, A. H. MacDonald, G. Shvets, Photonic topological insulators. *Nat. Mater.* **12**, 233–239 (2013).

4. L. H. Wu, X. Hu, Scheme for achieving a topological photonic crystal by using dielectric material. *Phys. Rev. Lett.* **114**, 223901 (2015).

5. T. Ma, G. Shvets, All-Si valley-Hall photonic topological insulator. *New J. Phys.* **18**, 025012 (2016).

6. T. Ma, G. Shvets, Scattering-free edge states between heterogeneous photonic topological insulators. *Phys. Rev. B* **95**, 165102 (2017).

7. J. W. Dong, X. D. Chen, H. Zhu, Y. Wang, X. Zhang, Valley photonic crystals for control of spin and topology. *Nat. Mater.* **16**, 298–302 (2017).

8. F. Gao, H. Xue, Z. Yang, K. Lai, Y. Yu, X. Lin, Y. Chong, G. Shvets, B. Zhang, Topologically protected refraction of robust kink states in valley photonic crystals. *Nat. Phys.* **14**, 140–144 (2017).

9. S. Barik, A. Karasahin, C. Flower, T. Cai, H. Miyake, W. DeGottardi, M. Hafezi, E. Waks, A topological quantum optics interface. *Science* **359**, 666–668 (2018).

10. N. Parappurath, F. Alpeggiani, L. Kuipers, E. Verhagen, Direct observation of topological edge states in silicon photonic crystals: Spin, dispersion, and chiral routing. *Sci. Adv.* **6**, eaaw4137 (2020).

11. P. Lodahl, S. Mahmoodian, S. Stobbe, A. Rauschenbeutel, P. Schneeweiss, J. Volz, H. Pichler, P. Zoller, Chiral quantum optics. *Nature* **541**, 473–480 (2017).

12. Y. Wu, C. Li, X. Hu, Y. Ao, Y. Zhao, Q. Gong, Applications of topological photonics in integrated photonic devices. *Adv. Opt. Mater.* **5**, 1700357 (2017).

13. W. Bogaerts, D. Perez, J. Capmany, D. A. B. Miller, J. Poon, D. Englund, F. Morichetti, A. Melloni, Programmable photonic circuits. *Nature* **586**, 207–216 (2020).

14. C. Han, M. Lee, S. Callard, C. Seassal, H. Jeon, Lasing at topological edge states in a photonic crystal l3 nanocavity dimer array. *Light. Sci. Appl.* **8**, 40 (2019).

15. Y. Zeng, U. Chattopadhyay, B. Zhu, B. Qiang, J. Li, Y. Jin, L. Li, A. G. Davies, E. H. Linfield, B. Zhang, Y. Chong, Q. J. Wang, Electrically pumped topological laser with valley edge modes. *Nature* **578**, 246–250 (2020).

16. L. Yang, G. Li, X. Gao, L. Lu, Topological-cavity surface-emitting laser. *Nat. Photonics* **16**, 279–283 (2022).

17. S. Arora, T. Bauer, R. Barczyk, E. Verhagen, L. Kuipers, Direct quantification of topological protection in symmetry-protected photonic edge states at telecom wavelengths. *Light. Sci. Appl.* **10**, 9 (2021).

18. U. K. Chettiar, A. R. Davoyan, N. Engheta, Hotspots from nonreciprocal surface waves. *Opt. Lett.* **39**, 1760–1763 (2014).

19. L. Shen, X. Zheng, X. Deng, Stopping terahertz radiation without backscattering over a broad band. *Opt. Express* **23**, 11790–11798 (2015).

20. K. L. Tsakmakidis, L. Shen, S. A. Schulz, X. Zheng, J. Upham, X. Deng, H. Altug, A. F. Vakakis, R. W. Boyd, Breaking lorentz reciprocity to overcome the time-bandwidth limit in physics and engineering. *Science* **356**, 1260–1264 (2017).

21. S. A. H. Gangaraj, F. Monticone, Do truly unidirectional surface plasmon-polaritons exist? *Optica* **6**, 1158–1165 (2019).

22. D. E. Fernandes, M. G. Silveirinha, Topological origin of electromagnetic energy sinks. *Phys. Rev. Appl.* **12**, 014021 (2019).

23. S. A. Mann, D. L. Sounas, A. Alù, Nonreciprocal cavities and the time–bandwidth limit. *Optica* **6**, 104–110 (2019).

24. S. Buddhiraju, Y. Shi, A. Song, C. Wojcik, M. Minkov, I. A. D. Williamson, A. Dutt, S. Fan, Absence of unidirectionally propagating surface plasmon-polaritons at nonreciprocal metal-dielectric interfaces. *Nat. Commun.* **11**, 674 (2020).

25. S. A. Hassani Gangaraj, B. Jin, C. Argyropoulos, F. Monticone, Broadband field enhancement and giant nonlinear effects in terminated unidirectional plasmonic waveguides. *Phys. Rev. Appl.* **14**, 054061 (2020).

26. S. A. Mann, A. Mekawy, A. Alù, Broadband field localization, density of states, and nonlinearity enhancement in nonreciprocal and topological hotspots. *Phys. Rev. Appl.* **15**, 034064 (2021).

27. Y. Li, Y. Yu, F. Liu, B. Zhang, G. Shvets, Topology-controlled photonic cavity based on the near-conservation of the valley degree of freedom. *Phys. Rev. Lett.* **125**, 213902 (2020).

28. N. Rotenberg, L. Kuipers, Mapping nanoscale light fields. *Nat. Photonics* **8**, 919–926 (2014).

29. X.-D. Chen, F.-L. Zhao, M. Chen, J.-W. Dong, Valley-contrasting physics in all-dielectric photonic crystals: Orbital angular momentum and topological propagation. *Phys. Rev. B* **96**, 020202 (2017).

30. C. A. Rosiek, G. Arregui, A. Vladimirova, M. Albrechtsen, B. Vosoughi Lahijani, R. E. Christiansen, S. Stobbe, Observation of strong backscattering in valley-Hall photonic topological interface modes. *Nat. Photonics* **17**, 386–392 (2023).

31. J. D. Joannopoulos, S. G. Johnson, J. N. Winn, R. D. Meade, *Photonic Crystals: Molding the Flow of Light* (Princeton Univ. Press, ed. 2, 2008).

32. C. P. Reardon, I. H. Rey, K. Welna, L. O'Faolain, T. F. Krauss, Fabrication and characterization of photonic crystal slow light waveguides and cavities. *J. Vis. Exp.* e50216 (2012).

33. M. I. Shalaev, W. Walasik, A. Tsukernik, Y. Xu, N. M. Litchinitser, Robust topologically protected transport in photonic crystals at telecommunication wavelengths. *Nat. Nanotechnol.* **14**, 31–34 (2019).

34. Y. Li, Y. Yu, K. Lai, Y. Han, F. Gao, B. Zhang, G. Shvets, Mode-selective single-dipole excitation and controlled routing of guided waves in a multi-mode topological waveguide. *Appl. Phys. Lett.* **120**, 221702 (2022).

35. G. W. Semenoff, Condensed-matter simulation of a three-dimensional anomaly. *Phys. Rev. Lett.* **53**, 2449–2452 (1984).
